# Immunolocalization of the 29 kDa *Schistosoma haematobium* species-specific antigen: a potential diagnostic marker for urinary schistosomiasis

**DOI:** 10.1186/s12879-015-0931-y

**Published:** 2015-04-26

**Authors:** Uri S Markakpo, George E Armah, Julius N Fobil, Richard H Asmah, Isaac Anim-Baidoo, Alfred K Dodoo, Parnor Madjitey, Edward E Essuman, Somei Kojima, Kwabena M Bosompem

**Affiliations:** Department of Biological, Environmental and Occupational Health Sciences, School of Public Health, College of Health Sciences, University of Ghana, P. O. Box LG13, Legon Accra, Ghana; Noguchi Memorial Institute for Medical Research, College of Health Sciences, University of Ghana, P. O. Box LG581, Legon Accra, Ghana; School of Allied Health Sciences, College of Health Sciences, University of Ghana, Accra, Ghana; Asian Centre for International Parasite Control, Mahidol University, Bankok, Thailand

**Keywords:** Schistosomiasis, *S. haematobium*, Antigen, MAb, Immunolocalization, Fluorescence

## Abstract

**Background:**

The 29 kDa *Schistosoma haematobium* species-specific antigen (ShSSA) is of remarkable interest in the diagnosis of urinary schistosomiasis although it had not been fully characterized.

**Method:**

To determine the biological importance of ShSSA in *S. haematobium* and pathogenesis of the disease, we immunolocalized ShSSA in schistosome eggshells, miracidia and adult worm sections using indirect fluorescent antibody test (IFAT).

**Results:**

ShSSA was strongly immunolocalized in the schistosome eggshells, selective regions of the miracidia body and walls of internal organs such as oviduct, ovary, vitelline duct and gut of the adult worm.

**Conclusion:**

The strong immunolocalization of ShSSA in schistosome eggshells and adult worm internal organs suggests that the antigens involved in the pathogenesis of urinary schistosomiasis could have originated from the eggs and adult worms of the parasite.

The findings also indicate that ShSSA may play a mechanical protective role in the survival of the parasite.

## Background

Schistosomiasis caused by trematode parasites of the genus *Schistosoma* is still a very important waterborne disease affecting humans today and yet considered as a neglected tropical disease because of the misunderstanding of the burden of the disease by policy makers. Schistosome infection may cause severe pathology of the liver, spleen, kidneys, bladder and urinogenital tract, and is responsible for high morbidity in endemic areas with an estimated loss of 1.76 million DALYs [1].

Schistosome antigens are reported to be partly involved in the pathology of schistosomiasis [2]. Several schistosome antigens such as the variant forms of glutathione S-transferase (P28/GST) and the 97 kDa paramyosin (Sm97) have been studied with most of them derived from *S. mansoni* and *S. japonicum* [3].

Characterization of schistosome antigens identified by monoclonal antibodies (MoAbs) could enhance schistosomiasis control for two main reasons. Firstly, such antigens may carry specific epitopes serving as targets for immune attack and are therefore potential candidates for vaccine production [4,5]. Secondly, where the antigen has diagnostic potential, it may be explored to improve diagnosis and provide useful information on evolution and classification of schistosomes. Identification and characterization of more schistosome antigens, especially from *S. haematobium* are therefore necessary for improving diagnosis and treatment outcomes.

A 29 kDa*S. haematobium* species-specific antigen (ShSSA) was identified in both Ghanaian and Egyptian strains of the parasite [6,7]. Even though a monoclonal antibody (**MAb**) to ShSSA has been successfully used in a field applicable dipstick for diagnosis of urinary schistosomiasis [8,9] ShSSA has not been fully characterized. Immunolocalization to characterize this antigen at the morphological and ultrastructural levels in *S. haematobium* will provide answers to critical questions about the use of the antigen in estimating infection intensity. Furthermore, immunolocalization of the antigen will provide data on its role in the survival of the parasite and significance in its taxonomy [10]. A major objective of this study, therefore, was to immunolocalize ShSSA in all life-cycle stages of *S. haematobium*. In addition, in order to ascertain the suitability of infected urine samples and parasite eggs for immunolocalization studies on ShSSA, this study was conducted with a view to determine the reactivity of **MAb** to crude antigens from eggs and urine of study subjects. Finally, to identify urine samples that can provide adequate amount of parasite eggs for generation of *S. haematobium* life-cycle stages and crude antigen extracts, this study was conducted to determine the sensitivity and specificity of microscopy or **MAb** dipstick test at detecting parasite eggs or antigens from the urine of study subjects.

## Methods

### Study design and population

The study was a purposive cross sectional study involving elementary school pupils who answered yes to whether or not they have any of the signs and symptoms of urinary schistosomiasis**.**

The *S. haematobium* species-specific **MAb** required for detection of the 29 kDa antigen was purified and the reactivity confirmed. Active **MAb** fractions were utilized for the urinary schistosomiasis **MAb** dipstick assay (USDA), microplate enzyme-linked immunosorbent assay (ELISA) and indirect fluorescent antibody test (IFAT). Urine samples for the study were collected from a total of 292 elementary school pupils from two villages, Kwashikumahman (n = 190) and Kojo Ashong (n = 102), hyperendemic for urinary schistosomiasis [11]. Aliquots of urine samples from subjects showing urinary schistosomiasis symptoms, haematuria and dysuria, were tested for *S. haematobium* antigens and eggs using USDA and microscopy respectively. Schistosome eggs were isolated from urine samples with >100 eggs/10 ml of urine for soluble egg antigen preparation, generation of parasite stages and for immunolocalization.

### Study area

The study was conducted at Kojo Ashong and Kwashikumahman in the Greater Accra Region of Ghana. These villages are located on 5°43'N, 0°23.5'E and 5°43'N, 0°21.5'E, respectively. The vegetation along the banks of the slow flowing Densu River and Dobro stream, running at the outskirts of the villages, comprises mainly grassland and a few trees. The weedy river and stream banks contain decomposing plant leaves and twigs infested with urinary schistosomiasis vector snails, *Bulinus globosus*. The Densu River and the Dobro stream constitute the principal sources of water for domestic use and transmission of urinary schistosomiasis.

### Ethical consideration

Study subjects were elementary school pupils assigned by the Ghana Health Service, Ministry of Education and Noguchi Memorial Institute for Medical Research for schistosomiasis examination and treatment. Subjects were recruited if they discharged urine with blood and/ or pain. Informed consent was obtained from Education Authorities and parents and/or guardians of pupils before recruitment. Infected children were treated with 40mg/kg body weight of praziquantel (Merck KGaA, Darmstadt, Germany) as recommended by WHO [12]. Approval for this study was given by the Noguchi Memorial Institute for Medical Research Institutional Review Board, referenced *NMIMR-IRB CPN 042/06-07 rev 2008.*

### Collection and analysis of urine specimens

#### Field procedures

##### Collection and handling of urine samples

Twenty to 100ml of fresh clean catch urine was collected from each of 292 school pupils in the period between 11:00 and 14.00hours Greenwich Mean Time (GMT) into a 200ml urine container. The samples were then transported to the laboratory at the Noguchi Memorial Institute for Medical Research within 1hour on ice in ice-chest.

### Laboratory procedures

#### Examination of urine samples for parasite antigens and eggs

Within 3hours after arrival at the laboratory urine samples were tested individually for *S. haematobium* antigen by **MAb** dipstick as described elsewhere [8,9,11]. Also, 10ml of the urine was filtered through a 25mm Nucleopore filter (12μm pore size) [11] to determine parasite density. The rest of the urine was centrifuged at 1,290 X g to isolate *S. haematobium* eggs.

### Generation of *S. haematobium* parasite life-cycle stages

*S. haematobium* eggs were isolated by centrifugation and sedimentation as described by Bosompem and others [13] and then cleaned by density centrifugation through ficoll-paque™ (GE Healthcare Life Sciences, Buckinghamshire, UK). They were subsequently hatched into miracidia by exposure to clean aged tap water and light as described by Huyse and others [14]. Some of the miracidia were used to infect *Bulinus* snails (five miracidia/ snail) to generate cercariae as described elsewhere [15,16]. Some of the live cercariae were transformed into schostosomula by vortexing (Ikemoto Rikakogyo Co. Ltd., Japan) as described by Ramalho-Pinto and others [17] for 20min. Some cercariae were also used to infect BALB/c mice to generate adult worms [6,15]. Fractions of the eggs, miracidia, cercariae, schistosomula and adult worms were respectively homogenized by sonication [15] to prepare crude antigens or treated with fixatives for immunolocalization studies.

### *S. haematobium* parasite stages and fixatives for immunolocalization

*S. haematobium* parasite stages were fixed for immunolocalization according to the method described elsewhere [18] with modification.

Washed *S. haematobium* eggshells, miracidia, cercariae, schistosomula and adult worms were suspended in PBS containing different concentrations of fixatives namely, paraformaldehyde, glutaraldehyde, Karnovsky’s fixative, methanol, ethanol and acetone, and incubated at 4°C for 5 min, 30 min, 60 min, 90 min and overnight (12 hrs). The concentrations of the various fixatives tested were, 0.1, 0.25, 0.5, 1.0, 1.5, 2.0, 2.5, 3.0, 4.0, 5.0, 10 and 100%. Fixed specimens were washed four times with PBS by centrifugation at 16,000 xg for 10 min at 4°C, stored in PBS at 4°C and analyzed by a preliminary IFAT to determine and select the best fixatives and conditions for immunolocalization studies.

### Preparation of *S. haematobium* adult worms for (IFAT)

Adult worms were prepared for IFAT as described elsewhere [19-21] with modification. Fixed *S. haematobium* adult worms were incubated for 5mins in saturated picric acid to colour the specimen and enhance visualization. The worms were dehydrated stepwise by transferring them into ethanol (85% for 10 min, 95, 100 and 100%, each for 20 min) and then 100% chloroform (3 times, each for 10 min). The worms were transferred into molten paraffin wax at 56°C (2 times for 10 min each) to fill open cavities with wax, embedded in wax and then cut into 4 μm sections with a microtome (Yamato Koki Individuals. Co., Ltd., Tokyo, Japan.). Worm sections on glass microscope slides were deparaffinized by heating at 50°C for 2 hr and then washed twice in xylene (5 min/wash). The worms were then re-hydrated stepwise in 100, 100, 95 and 90% ethanol (5 min/step). Finally, the worm sections were rinsed, 5 min each, under running distilled water and in PBS.

### Monoclonal antibody (MAb)

The IgG1**MAb** used in this study was generated by immunizing BALB/c mice with antigens extracted from *S. haematobium* infected human urine [6,7]. This antibody did not cross-react with *Necator americanus* (hookworm) egg antigens in micro-plate ELISA, and could bind ShSSA, from the eggs of both Ghanaian and Egyptian strains of *S. haematobium* and infected human urine [6-8]. The antibody in culture supernatant was concentrated ten-fold by Amicon filtration or by precipitation with 50% (v/v) ammonium sulphate [(NH_4_)_2_SO_4_] and then purified by ion-exchange chromatography. Microplate ELISA was used to determine the reactivity of this **MAb** to ShSSA in infected human urine and homogenates of parasite stages as described earlier [6].

### IFAT procedure

IFAT was conducted on the prepared parasite stages as described elsewhere [19,22] with modification. Fixed eggshells, miracidia, cercariae and schistosomula in suspension were coated, 20–30 specimens/well, onto multi-well IFA slides by heating briefly over a Bunsen flame. Slides coated with worm sections or other parasite stages were immersed briefly in a destaining jar containing 0.2 M Phosphate Buffered Saline (PBS, pH 7.2) to wash off loosely bound materials. Excess PBS was blotted by touching the edge of the slides with filter paper after which the wells were incubated with Sh2/15.F, positive control (immune) sera or negative control (free medium) at 20 μl/well for 1 hr at room temperature. Primary antibodies, Sh2/15.F and immune sera were used at 1:40 and 1:50 dilution, respectively. The slides were washed twice (5mins per wash) with PBS and then incubated for 30 min (20 μl/well) with secondary antibody reagent [goat-anti-mouse IgG conjugated with fluorescein isothiocyanate (FITC) (Sigma Immuno Chemicals, St. Louis, MO) and 0.01% trypan blue (counter stain), all in PBS]. After incubation the slides were washed four times in PBS, blotted and then mounted in 50% glycerol (Sigma-Aldrich Co. Ltd.-Gillingham-Dorset, UK) in PBS. The specimens were observed using a flourescent microscope (Olympus Optical Co. Ltd., Japan) at x120 magnification.

## Results

### Reactivity of purified MAb fractions

Table 1 summarizes the reactivity of different fractions of **MAb** to crude antigens from *S. haematobium* parasite stages and infected human urine as determined by micro-plate ELISA. The results show that **MAb** was reactive to crude antigens from all life-cycle stages of the parasite and infected human urine.

**Table 1:** **Reactivity of purified MAb.F fractions with crude antigens from** ***S. haematobium*** **parasite stages and infected human urine**

**Antibody fraction**	**Crude antigens**					
	**Egg lysate**	**P**_**2**_ J*	**Miraci-dium**	**Cercaria**	**Schistoso-mulum**	**Adult worm**
Amicon concentrated	2+	3+	>+	+	<+	>+
Ion-exchange purified	2+	2+	+	+	<+	+
Precipitated by 50% (NH)_2_SO_4_ solution	3+	2+	>+	+	<+	>+
Immunized mouse serum^α^	3+	3+	>+	+	<+	>+
Normal mouse serum^β^	-	-	-	-	-	-
Antibody-free culture medium^ω^	-	-	-	-	-	-

*Crude antigens extracted from *S. haematobium* infected human urine.

α Positive control sample.

β Negative control sample.

ω Background blank sample.

+ Positive reaction.

- Negative reaction.

> + Stronger than positive, but weaker than 2+.

<+ Weak reaction or trace.

### Prevalence of Urinary Schistosomiasis

Table 2, summarizes urinary schistosomiasis prevalence both at Kojo Ashong and Kwashikumaman as determined by microscopy and **MAb**-dipstick, USDA. Of the 292 individuals interviewed verbally for the presence or absence of urinary schistosomiasis, 58.56% (171/292) answered yes. Also, USDA detected *S. haematobium* antigens in more (55.82%) of the 292 subjects than microscopy (*p* < 0.01). The analysis showed that the urinary schistosomiasis prevalence estimated by microscopy in Kwashikumaman (51.58%) (98/190) was higher (*p* < 0.01) than that of Kojo Ashong (29.41%) (30/102). A similar trend was found using the USDA (*p* < 0.01). All individuals who were *S. haematobium* egg positive also tested positive for parasite antigens by the USDA.

**Table 2:** **Prevalence of urinary schistosomiasis in the study communities as determined by microscopy and dipstick assay**

**Community**	**Number tested**	**Microscopy+ve (%)**	**Dipstick+ve (%)**	*Sensitivity(%)	*Specificity (%)
K A	102	30 (29.41)	42 (41.18)	100	75.00
K M	190	98 (51.58)	121 (63.68)	100	83.33
Total	292	128 (43.84)	163 (55.82)	100	78.66

K A Kojo Ashong.

K M Kwashikumahman.

*Relative sensitivity and specificity based on microscopy as gold standard test.

### Evaluation and selection of fixatives for immunolocalization

Table 3 summarizes the results of the experiment to determine the suitability of six fixatives (paraformaldehyde, glutaraldehyde, Karnovsky’s fixative, acetone, methanol and ethanol) for processing *S. haematobium* parasite stages for immunolocalization. As shown, 2.0% Karnovsky’s fixative (2% paraformaldehyde plus 2% glutaraldehyde) applied for 90 min at 4°C, produced the highest positive fluorescence with a weak background staining as compared to the other fixatives. Consequently, this fixative at the application conditions was used to process the specimens for immunolocalization of the diagnostic antigen.

**Table 3:** **a & b: evaluation of different fixatives for processing** ***S. haematobium*** **(Sh) parasite stages for immunolocalization (IL)**

**Table 3a**							
**Type of fixative**	**Duration of treatment of Sh parasite stages**	**Concentration of fixative**					
		**0.10**	**0.25**	**0.50**	**1.00**	**1.50**	**2.00**
Karnovsky’s fixative	90 min					+ (+)	3+ (±)
	12 hr					+ (+)	2+ (±)
Paraform-aldehyde	90 min						
	12 hr						
Glutar-aldehyde	90 min			3 + (2+)	3 + (2+)	2 + (2+)	+ (±)
	12 hr			3 + (2+)	2 + (2+)	2 + (2+)	± (±)
Methanol	90 min	+ (±)	± (±)				
	12 hr	+ (±)	± (±)				
Ethanol	90 min	± (±)	+ (±)				
	12 hr	+ (±)	± (±)				
Acetone	5 min						
	90 min	2+ (−)	2+ (−)				
	12 hr	2+ (−)	2+ (−)				
**Table 3b**							
**Type of fixative**	**Duration of treatment of Sh parasite stages**	**Concentration of fixative**					
		**2.50**	**3.00**	**4.00**	**5.00**	**10.00**	**100.00**
Karnovsky’s fixative	90 min	+ (+)					
	12 hr	+ (+)					
Paraform-aldehyde	90 min		± (−)	± (−)			
	12 hr		+ (−)	+ (−)			
Glutar-aldehyde	90 min	+ (±)	± (±)				
	12 hr	± (±)	± (±)				
Methanol	90 min						
	12 hr						
Ethanol	90 min						
	12 hr						
Acetone	5 min						2+ (−)
	90 min						
	12 hr						

Results in parenthesis, ( ), indicate the intensity of background staining.

± Indicates weak fluorescence.

Blank spaces on the table represent negative fluorescence and background staining results. Except for 100% acetone which gave positive fluorescence at 5 min of fixation, all the reagents at all the concentrations gave negative fluorescence and background staining at 5, 30 and 60 min of fixation, and were not represented on the table.

#### Immunolocalization of the 29 kDa *S. haematobium* antigen using IFAT

**Figure 1:**
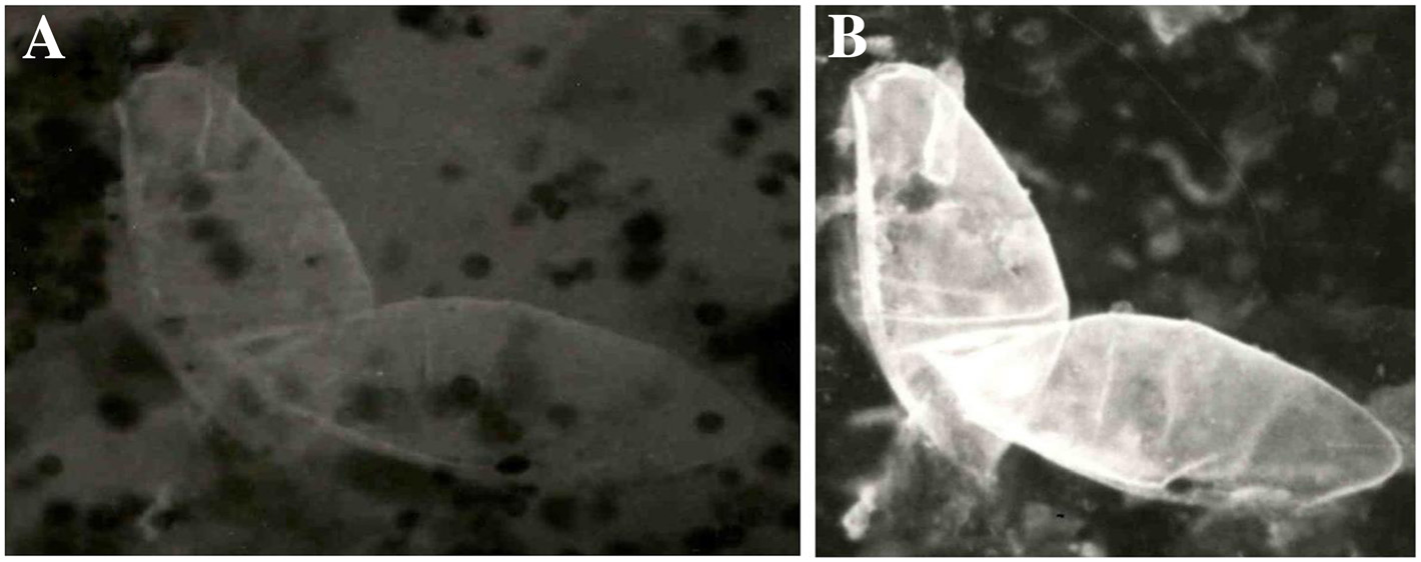
Immunolocalization of the 29 kDa *antigen* in *S. haematobium egg shell by immunofluorescence.* **(A)** and **(B)** are micrographs of *S. haematobium* eggshells under x120 magnification, showing strong fluorescence (immunolocalization of ShSSA*) in the outer membrane and inner lining of the shells following incubation with **MAb** and anti-mouse-FITC conjugate antibody. **(A)** Under ordinary light **(B)** under fluorescent light (*) 29 kDa *Schistosoma haematobium* species-specific (diagnostic) antigen.

**Figure 2:**
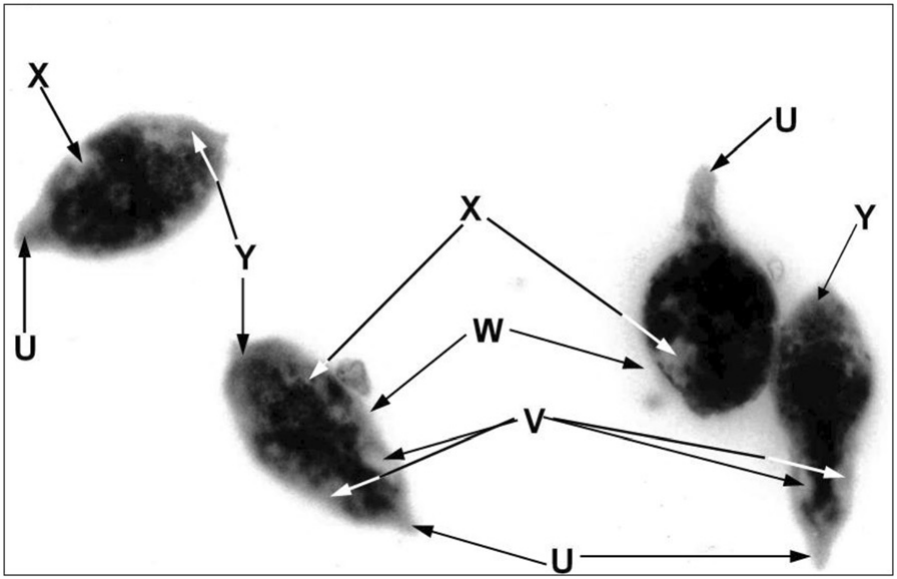
Immunolocalization of the 29 kDa *antigen* in *S. haematobium miracidia by immunofluorescence.* Figure is a micrograph of *S. haematobium* miracidia, under x120 magnification, showing immunolocalization of ShSSA in selective regions of the body, following incubation with Sh2/15.F MAb and anti-mouse-FITC conjugate antibody. U) Tail, V) Basal regions of lateral sides of the body trunk, W) Cilia, X) Patches on the dorsal surface of the tegument, Y) Anterior region of the body.

**Figure 3:**
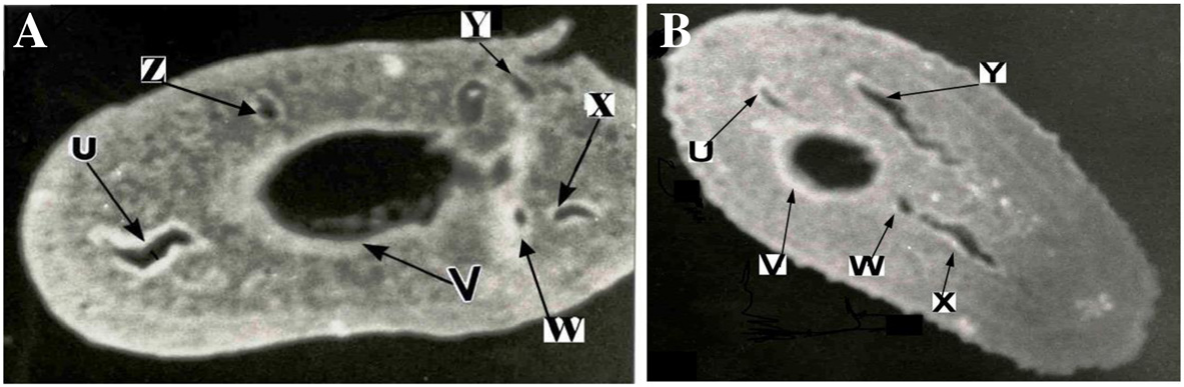
Immunolocalization of the 29 kDa *antigen* in *S. haematobium adult worm cross-sections by immunofluorescence.* **(A)** and **(B)** are micrographs of cross-sections from two different *S. haematobium* adult worms under x120 magnification showing strong immunolocalization of ShSSA in the walls of various internal organs such as oviduct, ovary, vitelline duct and digestive caeca, labelled U, V, W, X, Y and Z, following incubation with Sh2/15.F **MAb** and anti-mouse-FITC conjugate antibody.

The 29 kDa *S. haematobium* diagnostic antigen, ShSSA, was immunolocalized to the scleroproteic eggshell (Figure 1). However, the inner wall of the eggshell (vitelline membrane) showed more intense fluorescence in some specimens. As shown in Figure 2, the antigen was also immunolocalized mostly to the cilia and patches on the dorsal surface of the tegument of the miracidia. In the adult worm, ShSSA was immunolocalized in the entire section. However, the antigen appeared to be concentrated in the inner lining of internal organs such as the ovary and gut (Figure 3).

## Discussion

The aim of this study was to immunolocalize the 29 kDa *Schistosoma haematobium* species-specific antigen (ShSSA) in all the life-cycle stages of the parasite. Even though a monoclonal antibody (Sh2/15.F) against this antigen has enabled it to be identified in a field-applicable dipstick test as a potential diagnostic marker for the disease [8,9,11], ShSSA has not been fully studied. Immunolocalization of ShSSA at the morphological and ultrastructural levels in the parasite, therefore, is of paramount importance to provide answers to critical questions about the relative abundance of the antigen in the parasite stages and its applicability in estimating infection intensity. Also, immunolocalization studies on this antigen will provide data on the possible role of ShSSA in the survival of the parasite. Lastly, immunolocalization of this antigen will provide information on the stage of the parasite life-cycle to concentrate efforts on for studies on this antigen. To achieve this aim, therefore, we isolated parasite eggs from the urine of infected school-aged children from which we generated the various life-cycle stages of the parasite [6,13-17]. Immunofluorescence assay was then used to localize the 29 kDa Species-specific antigen in the various life-cycle stages of the parasite [18-22].

From this study, the 29 kDa antigen (ShSSA) was detected in all the life-cycle stages of *S. haematobium* and in the urine of infected individuals. To our knowledge, this is the first study to immunolocalize a species-specific potential diagnostic marker antigen in the various life-cycle stages of the *S. haematobium* parasite.

Immunolocalization of proteins provides answers to critical questions concerning the roles of specific antigens in classification and survival of parasites [10]. For example, immunolocalization studies confirmed paramyosin as a muscle protein and contributed to the understanding of its multiple functions within the schistosome [10]. In this study, the ShSSA present in both Ghanaian and Egyptian strains of *S. haematobium* [7] was immunolocalized in the eggshells, miracidia and adult worm. This observation coupled with the detection of ShSSA in homogenates of *S. haematobium* adult worm, eggs, miracidia, cercariae and schistosomula by microplate ELISA and USDA, indicated that ShSSA may be distributed in all the life-cycle stages of the parasite.

Also, the identification of ShSSA in strains of the parasite from Egypt and different regions of Ghana [7-9] suggested that this antigen has a wide geographic distribution. In addition the wide distribution of this antigen in *S. haematobium* both stage-wise and geographically *vis-a-vis* its absence in *S. mansoni* and *N. americanus* [7-9], emphasizes its possible specificity to *S. haematobium* and potential usefulness in taxonomic classification of the parasite and diagnosis of urinary schistosomiasis.

Labelling of ShSSA in the eggshells and miracidia confirmed the presence of the antigen in the *S. haematobium* eggs. The *S. haematobium* eggshell is scleroproteic and has sub-microscopic pores through which antigens are excreted [23-27]. It is therefore, possible that the antigen immunolocalized is a structural component of the eggshell, had diffused from the miracidia into the shell, or both.

In addition, the immunolocalization of ShSSA in the eggshell, tegument and cilia of miracidia and walls of internal organs such as ovary, vitelline duct and gut of the adult worm, suggests that, possibly, this protein has a muscular function in the parasite. Further studies are however required to ascertain this observation. Furthermore, the localization of ShSSA in both eggs and adult worms of *S. haematobium* suggests that the antigen detected in infected persons may originate from both stages of the parasite, since these are the stages found in humans. Hence, it may be difficult but interesting to use measured antigen levels to estimate egg or worm burden.

High relative sensitivity (99.1%) and specificity (98.3%) were reported for USDA in earlier studies [11], however, in this work, the specificity (78.66%) of the **MAb**-dipstick compared to microscopy as a gold standard test was significantly lower (*p* < 0.01) whilst the sensitivities were the same (100%). The rather low relative specificity recorded by USDA in this study could be attributable to either a low sensitivity of the microscopic technique or high false positivity rate of the USDA. The first explanation is more likely to be correct because, Bosompem *et al*. [11] re-examined urine samples with low egg counts, which readily tested positive by USDA, seven times more before confirming their positivity by microscopy. The higher (*p* < 0.01) prevalence of urinary schistosomiasis obtained by USDA compared to microscopy was therefore not surprising. One limitation of this study is the inability to repeat testing of the samples to ascertain parasite infectivity rates.

## Conclusions

In conclusion, this study has confirmed the suitability of the USDA for detecting *S. haematobium* infections in humans. The 29 kDa antigen was found in all the life-cycle stages of *S. haematobium* and in the urine of infected individuals. The 2% Karnovsky’s fixative was shown to be suitable for immunolocalization studies on ShSSA. The presence of ShSSA in both eggs and adult worms of *S. haematobium* suggests that the antigen detected in infected persons may originate from both stages of the parasite.

### Recommendations

It is recommended that further immunolocalization studies be conducted on the 29 kDa *S. haematobium* species-specific antigen at the ultrastructural level by electron microscopy to elucidate the function(s) of this diagnostic antigen in the various stages of *S. haematobium.*
